# Supplementary material for: High performance current and spin diode of atomic carbon chain between transversely symmetric ribbon electrodes
Source: Sci Rep. 2014 Aug 21;4:6157. doi: 10.1038/srep06157 (PMC4139955; doi:10.1038/srep06157)
Supplement: Supplementary Information — High performance current and spin diode of atomic carbon chain between transversely symmetric ribbon electrodes [file srep06157-s1.doc]

**High performance current and spin** **diode of atomic carbon chain between transversely symmetric ribbon electrodes**

Yao-Jun Dong^1^, Xue-Feng Wang^1, 2, *^, Shuo-Wang Yang^3^, Xue-Mei Wu^1, 2^

**Supplementary information**

**Supplementary Figure S1**

Figure S1. The calculated bond lengths (in unit of Å) of (a) B-doped and (b) N-doped 6-ZGNR.

The geometry of 6-ZGNRs doped by B or N atoms on both edge sides have been optimized until the force felt on each atom is less than 0.02 eV/Å. The bond lengths in optimized cells are shown in Figure S1.

**Supplementary Figure S2**


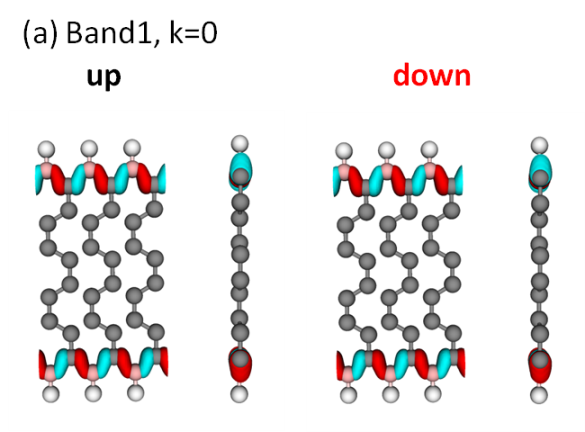

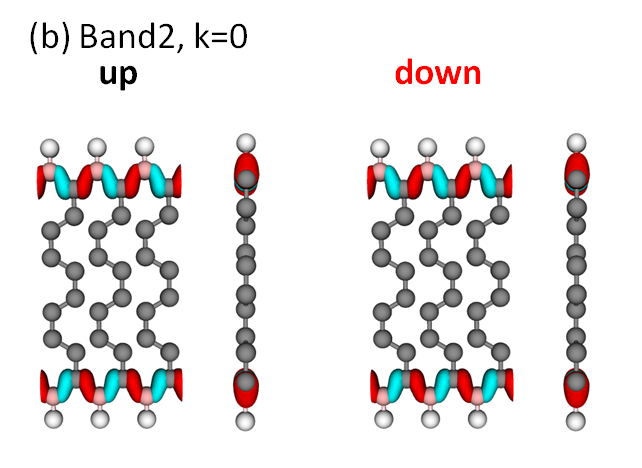

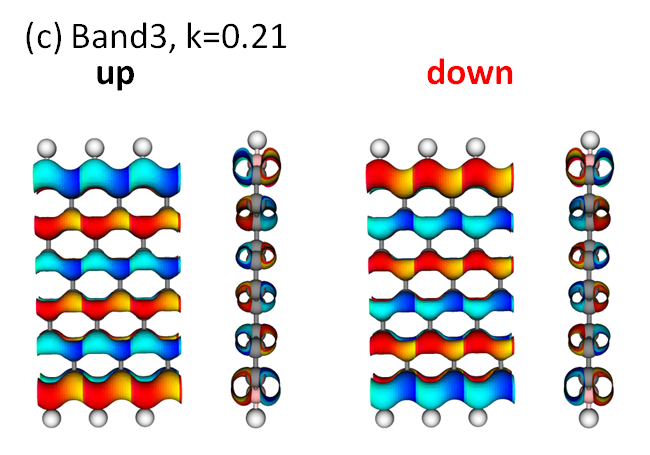

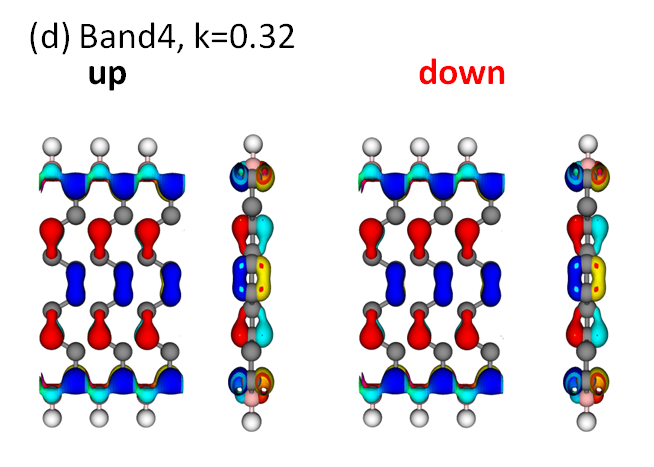

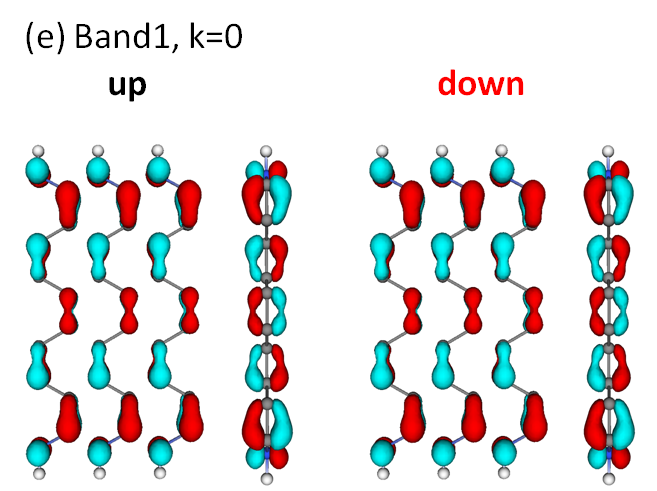

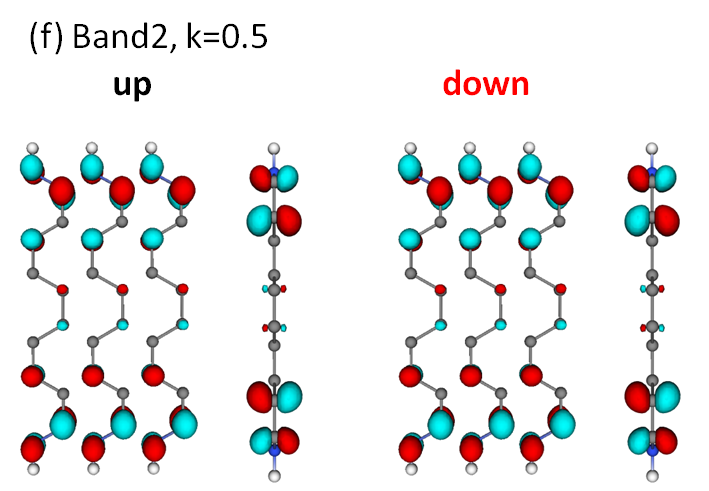

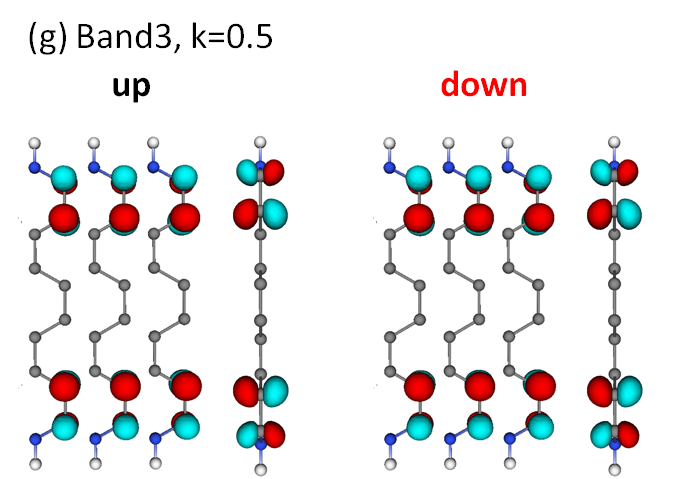


Figure S2. The wave function isosurfaces of the spin-up and spin-down electrons for Bands 1-4 in B-doped 6-ZGNRs (see Figure 3(d) in the main text) are plotted in (a)-(d), respectively and those for Bands 1-3 in N-doped 6-ZGNRs [see Figure 4(d) in the main text] are plotted in (e)-(g).

The electronic wave function isosurfaces of Bands 1-4 of B-doped 6-ZGNRs are given in Figure S2 (a)-(d), respectively. Bands 1 and 2 have characteristics of σ bonding, the Band 3 has characteristics of π bonding, and Band 4 has characteristics of π* bonding. Figure S2 (e)-(g) are the wave function isosurfaces of Bands 1-3 of N atomic doped 6ZGNRs, respectively. Bands 1 and 3 exhibit π* characteristics, while the Band 2 is π characteristic.
